# Hodgkin Lymphoma after Disseminated *Mycobacterium genavense* Infection, Germany

**DOI:** 10.3201/eid2807.220425

**Published:** 2022-07

**Authors:** Janina Trauth, Thomas Discher, Moritz Fritzenwanker, Can Imirzalioglu, Tobias Arnold, Dagmar Steiner, Elvira Richter, Laura Crisponi, Bodo Grimbacher, Susanne Herold

**Affiliations:** Justus Liebig University, Giessen, Germany (J. Trauth, T. Discher, M. Frizenwanker, C. Imirzalioglu, S. Herold);; University Hospital Gießen, Gießen (T. Arnold, D. Steiner);; MVZ Labor Doctor Limbach and Kollegen, Heidelberg, Germany (E. Richter);; Medical Center–University of Freiburg, Freiburg, Germany (L. Crisponi, B. Grimbacher)

**Keywords:** Hodgkin lymphoma, *Mycobacterium genavense*, nontuberculous *Mycobacterium*, cervical lymphadenopathy, zoonoses, Germany, tuberculosis and other mycobacteria

## Abstract

*Mycobacterium genavense* infection, a rare nontuberculous mycobacteria infection, occurs in heavily immunocompromised patients (i.e., those with advanced HIV disease, genetic disorders, or acquired immunologic disorders and those undergoing immunosuppressive therapy). We report a case of disseminated *M. genavense* infection preceding Hodgkin lymphoma in a patient without obvious risk factors for this infection.

*Mycobacterium genavense* was first described in 1992 in HIV-positive patients with low CD4 counts and disseminated mycobacterial disease (*1*). Since the 2000s, additional risk factors for this bacterial infection became known (e.g., solid organ transplantation, hematopoietic stem cell transplantation, Epstein-Barr virus–associated lymphoproliferative disorder, neutralizing anti–interferon γ autoantibodies, adenosine deaminase deficiency, nuclear factor κB1 deficiency) (*2*,*3*). Clinical manifestations of *M. genavense* commonly involve blood and lymph nodes but can include the gastrointestinal tract, spleen, liver, and bone marrow; pneumonia, prosthetic joint infection, endobronchial mass, and brain mass have also been described.

A previously healthy 23-year-old woman sought medical treatment at University Hospital Gießen (Gießen, Germany) for progressive cervical lymphadenopathy (Figure, panel A) and fever originating 4 months prior. A professional animal keeper, she had no history of previous infections or autoimmune disease, an unremarkable family history, and no travel outside of Europe; her tattoos showed no signs of irritation. She experienced gender dysphoria and used masculinizing hormone therapy (testosterone). We excluded common causes of cervical lymphadenopathy (e.g., HIV, tuberculosis, bacterial abscess, Epstein-Barr virus, lymphoma, toxoplasmosis, bartonellosis, and syphilis), but the extensive lymphadenopathy pointed to a severe disease (Figure, panel B, E). Multiple conglomerate, necrotizing mediastinal lymph nodes resulted in a tracheo-esophageal fistula (Figure, panel C), which required esophageal stenting.

Cervical lymph nodes showed a necrotizing, giant cell–containing inflammatory reaction. We detected acid-fast bacteria on microscopic examination and subsequently identified it as *M. genavense* by using broad-range 16S-rDNA PCR and Sanger sequencing of the resulting amplicon (Appendix). In blood and bone marrow, we detected no mycobacteria. From culture on solid medium and mycobacteria growth indicator tube, we were unable to recover outgrowth. *M. genavense* cannot be cultivated in routine liquid and solid media (Löwenstein–Jensen and Stonebrink) but requires special supplementation for recovery on culture (Middlebrook 7H11 agar [ThermoFisher, https://www.thermofisher.com] supplemented with mycobactin J) and an incubation period >100 days. Standardized susceptibility testing is not available (*4*).

For this nontuberculous mycobacteria (NTM) disease, diagnostic criteria are ill defined and no treatment guidelines are established. Reported case-patients are treated with a 2- to 4-drug regimen, including mostly macrolides, rifampin, ethambutol, and amikacin or fluroquinolones. The regimen for this patient consisted of clarithromycin, rifabutin, ethambutol, and temporary add-on doses of levofloxacin, amikacin, clofazimine, or bedaquiline. During the ensuing months, the wounds and tracheo-esophageal fistula slowly healed (Figure, panel D), and imaging showed decreased uptake (Figure, panel F).

**Figure:**
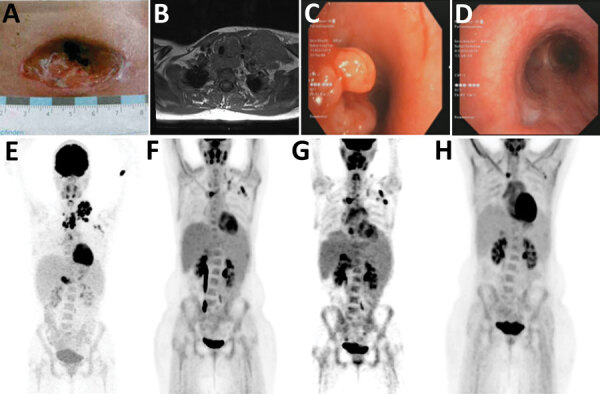
Clinical manifestations and radiologic findings in the course of disease in a 23-year-old woman with disseminated *M. genavense* infection preceding Hodgkin lymphoma, Germany. A) Cervical wound after initial lymph node extirpation. B) Magnetic resonance imaging at the time of initial evaluation. C) Endobronchial view of tracheo-esophageal fistula before positioning of a stent. D) Endobronchial view of the prior tracheo-esophageal fistula after treatment. Whitish scar tissue is seen at the bottom left. E) ^18^F-FDG-PET scan at initial evaluation (**maximum intensity projection)**. Cervical lymph node mass is seen, with no pathologic uptake in the abdomen. F) ^18^F-FDG-PET scan after 6 months of antibiotic treatment showing reduced uptake. G) ^18^F-FDG-PET scan shortly before Hodgkin lymphoma was diagnosed showing new hepatosplenomegaly and lymphadenopathy. H) ^18^F-FDG-PET scan after antibiotic and chemotherapy without pathologic enhancement.

As a professional pet keeper, the patient had close contact with domestic animals, including birds. Zoonotic transmission of *M. genavense* has not been well described (*5*), but it does pose a potential risk for susceptible hosts. Because a predisposing risk factor for the patient’s NTM disease had not been identified, we ruled out several conditions: acquired immunodeficiency, idiopathic CD4 lymphocytopenia, Mendelian susceptibility to mycobacterial disease, and neutralizing anti–interferon γ autoantibodies or a defect in the (proximal) interferon γ receptor signaling pathway (data not shown). A targeted gene panel with a focused analysis on 810 genes associated with immune and blood disorders did not identify a genetic variant that could alone explain the phenotype; however, we detected several rare variants (Appendix). 

After 11 months of antibiotic therapy, an ^18^F-FDG-PET scan revealed new lymphadenopathy and splenomegaly (Figure, panel G). CD4-to-CD8 ratio dropped from 1.7 to 1.0, and we found new low-level EBV viremia (350 copies/mL). On the basis of new tissue samples from mediastinal lymph nodes, we diagnosed classical Hodgkin lymphoma (HL [mixed type]) stage IV. Mycobacterial PCR was negative in all these samples and, retrospectively, all previous samples were tumor-free. Six cycles of chemotherapy (brentuximab combined with doxorubicin, vinblastine, dacarbazine) were followed by 4 doses of nivolumab because of histologically confirmed mixed response. One year after treatment completion and cessation of antimycobacterial therapy, liquid biopsy and an ^18^F-FDG-PET scan showed complete remission and no signs of NTM infection (Figure, panel H).

In other reports of *M. genavense* infections related to lymphomas, patients acquired the infection during immunosuppressive therapy; however, in this patient, infection preceded HL. Genetic and environmental factors are relevant in the pathogenesis of HL (*6*) and in pathogenic pathways triggered by virus infections (e.g., HIV and Epstein-Barr virus) (*7*); bacterial antigen triggering has been implicated recently in early developmental stages of the disease (*8*). Other reports have discussed an increased risk for HL after tuberculosis infection (*9*) and HL associated with concomitant tuberculosis, leprosy, and *Mycobacterium avium* complex disease (*10*).

*M. genavense* remains a diagnostic challenge because standard media and incubation times do not yield bacterial growth, which can result in missed diagnoses. Research is needed to gain a clear understanding of the interplay of NTM and HL, specifically in regard to how mycobacterial antigens trigger pathogenic pathways during HL development and the role of HL in causing local immune escape mechanisms and immunologic imbalance resulting in susceptibility to infections.

In conclusion, we report a patient with disseminated *M. genavense* infection preceding HL who recovered after antimycobacterial therapy and first- and second-line chemotherapy. A zoonotic source of *M. genavense* infection is likely. Furthermore, because sex hormones affect immunity and testosterone is a susceptibility factor for mycobacterial disease, masculinizing hormone therapy could have contributed to susceptibility.

Appendix Additional information about Hodgkin lymphoma after disseminated *Mycobacterium* *genavense* infection, Germany.
