# Supplementary material for: Hodgkin Lymphoma after Disseminated Mycobacterium genavense Infection, Germany
Source: Emerg Infect Dis. 2022 Jul;28(7):1506–9. doi: 10.3201/eid2807.220425 (PMC9239878; doi:10.3201/eid2807.220425)
Supplement: Appendix — Additional information about Hodgkin lymphoma after disseminated Mycobacterium genavense infection, Germany. [file 22-0425-Techapp-s1.pdf]

Article DOI: http://doi.org/10.3201/eid2807.220425

Hodgkin Lymphoma after Disseminated *Mycobacterium genavense* Infection, Germany

Appendix

Experimental procedure and description of the applied methodology

Experimental procedure

The DNA sample was analyzed using Next Generation DNA Sequencing technology. First, it was subjected to a standard quality control procedure and subsequently enriched and processed into a DNA library using the custom CeGaT_BID2 capture design, focused on the analysis of 810 genes associated with Immune and Blood Disorders. Second, the resulting targeted gene panel (TGP) library was checked and sequenced in a NovaSeq 6000 instrument.

Bioinformatics analysis

Raw data (FASTQ files) were processed using a custom bioinformatic pipeline that includes quality control, read trimming, alignment, variant calling with the FreeBayes, SamTools and GATK algorithms, and variant annotation using the VEP software from Ensembl. Resulting datasets were imported into our in-house SQL database, which were used to analyze and filter out common variants present in the general population or variants with a low predicted impact on protein function. Before filtering, a total of 5114 variants against the reference human genome (Dec. 2013, GRCh38/hg38) were identified in the sample.

Results interpretation

Variant priorization and classification was done using in-house–developed algorithms. For this study, we mainly considered rare variants (allele frequency below 1% in control populations). We did not identify one genetic variant that could alone explain the phenotype for the patient; however, we detected several rare variants that were of interest:

1) A heterozygous missense variant in *WAS* (c.995T>C, p.Val332Ala), which in the literature has been associated with X-linked thrombocytopenia in 2 cases, 1 being a

female carrier with a fully inactivated normal chromosome (*1*,*2*). Typically, patients with X-linked thrombocytopenia do not have significant immunodeficiency, but they do have an increased risk of malignancy (*3*).

2) A heterozygous missense variant in *IL17RA* (c.2245G>A, p.Glu749Lys); biallelic mutations in this gene can cause Immunodeficiency 51, an autosomal recessive disease characterized by chronic mucocutaneous candidiasis.
3) A novel variant of uncertain significance in *MEFV* (c.835G>A, p.Ala279Thr).
4) A probably benign variant in *TACI* (c.659T>C, p.Val220Ala).

Description of the applied methodology

<u>Initial mycobacteria real-time PCR</u>: DNA extraction from lymph-node-tissue was performed by an automated extraction system using a tissue protocol (eMAG, bioMérieux, https://www.biomerieux.com). The resulting DNA-extract was submitted to real-time PCR using the commercially available Anyplex MTB/NTMe Real-time Assay (Seegene, https://www.seegene.com), which allows detection of either *Mycobacterium tuberculosis* or broad-range nontuberculous mycobacteria. This assay, however, yielded only negative results, which might be due to an insufficient DNA-extraction protocol or lack of coverage for *Mycobacterium genavense* by the applied nontuberculous mycobacteria primers in the assay.

<u>Broad-range 16S-rDNA PCR and amplicon sequencing</u>: DNA extraction from lymph-node-tissue samples was performed using the PureLink Pro 96 Genomic DNA Purification Kit (Invitrogen, Fisher Scientific, https://www.fishersci.com/). 16S-rDNA PCR from extract was performed as previously described (*4*,*5*) using primers 933F (5‘ GCA CAA GCG GTG GAG CAT GTG G 3‘) and 1407R (5‘ GAT GGG CGG TCT GTA CAA G 3‘). The amplification products were analyzed for an expected 474bp amplicon by gel electrophoresis with a horizontal 1.5% agarose gel for 1 hour at 150 V at room temperature in TBE running buffer and then purified using the MSB® Spin PCRapace Kit (Invitek, https://invitek-molecular.com). Purfied DNA underwent Sanger-sequencing at an external certified and quality-controlled service-provider (Eurofins, https://eurofins.com).

<u>PCR/sequencing positive control</u>: *Escherichia coli* ATCC 25922. Reads were preliminarily analyzed at http://www.sepsitest-blast.de/de/index.html, both forward and reverse

reads yielding *M. genavense* (identity 99.8%; alignment length 417 (100.0%); E-value = 0.0; Accession = X60070 and identity 99.8%; alignment length 420 (98.6%); E-value = 0.0; Accession = X60070).

To verify, reads were then analyzed with CLC Genomics Workbench (v 12.0), QIAGEN Aarhus A/S, assembled with tracy assemble (v 0.6.1) on Galaxy Europe Server (*6*,*7*). The consensus was analyzed with BLASTN at NCBI nucleotide database (*8*,*9*), yielding multiple alignments, the best hit being *M. genavense* strain 2289, E value 0.0, identity 99.79% (476/477), gaps 0/477, score 876 bits, sequence ID NR 029223.1. Lower percentage hits were associated with other mycobacterial species.

To further confirm results, original samples were sent to Limbach laboratory (Germany) for independent PCR and sequence analysis. A lymph node biopsy was obtained for mycobacterial culture, and PCR analysis was performed for mycobacterial DNA. The minced tissue was decontaminated using the NALC-NaOH procedure (*10*) for mycobacterial culture. The resulting sediment was divided—one part was used for smear microscopy and was inoculated into MGIT liquid media and placed onto solid Löwenstein and Stonebrink slants, and the second part was used for molecular analyses by preparing DNA using the Fluorolyse technology (Bruker HAIN, https://bruker.com). A specific nested amplification protocol targeting the mycobacterial 16S rRNA gene was performed using primers '285' and '264' for 35 cycles and nested primers 'B9' and '247' for an additional 12 cycles (*11*). The obtained amplicon was sequenced by sanger sequencing using primer 'B9', resulting in a sequence of 510 bp, which was compared using the Basic Local Alignment Search Tool (https://blast.ncbi.nlm.nih.gov/). *M. genavense* was identified as the causative agent with 100% sequence homology with accession number AF547928 representing the type strain. PCR-negative controls proved negative.

In both laboratories, the smear microscopy of the tissue showed acid-fast bacteria, and liquid and solid cultures remained negative despite prolonged incubation.

The results were confirmed by analyzing a second lymph node biopsy obtained 3.5 months afterward and were also confirmed by the German National Reference Center for Mycobacteria (Borstel, Germany).

References

1. Daza-Cajigal V, Martínez-Pomar N, Garcia-Alonso A, Heine-Suñer D, Torres S, Vega AK, et al. X-linked thrombocytopenia in a female with a complex familial pattern of X-chromosome inactivation. Blood Cells Mol Dis. 2013;51:125–9. PubMed https://doi.org/10.1016/j.bcmd.2013.04.004

2. Proust A, Guillet B, Picard C, de Saint Basile G, Pondarré C, Tamary H, et al. Detection of 28 novel mutations in the Wiskott-Aldrich syndrome and X-linked thrombocytopenia based on multiplex PCR. Blood Cells Mol Dis. 2007;39:102–6. PubMed https://doi.org/10.1016/j.bcmd.2007.02.007

3. Albert MH, Bittner TC, Nonoyama S, Notarangelo LD, Burns S, Imai K, et al. X-linked thrombocytopenia (XLT) due to WAS mutations: clinical characteristics, long-term outcome, and treatment options. Blood. 2010;115:3231–8. PubMed https://doi.org/10.1182/blood-2009-09-239087

4. Domann E, Hong G, Imirzalioglu C, Turschner S, Kühle J, Watzel C, et al. Culture-independent identification of pathogenic bacteria and polymicrobial infections in the genitourinary tract of renal transplant recipients. J Clin Microbiol. 2003;41:5500–10. PubMed https://doi.org/10.1128/JCM.41.12.5500-5510.2003

5. Imirzalioglu C, Hain T, Chakraborty T, Domann E. Hidden pathogens uncovered: metagenomic analysis of urinary tract infections. Andrologia. 2008;40:66–71. PubMed https://doi.org/10.1111/j.1439-0272.2007.00830.x

6) Rausch T, Fritz M H-Y., Untergasser A, Benes V. Tracy: basecalling, alignment, assembly and deconvolution of sanger chromatogram trace files. BMC Genomics. 2020;21:230. **PMID 32171249** https://doi.org/10.1186/s12864-020-6635-8

7. Afgan E, Baker D, Batut B, van den Beek M, Bouvier D, Čech M, et al. The Galaxy platform for accessible, reproducible and collaborative biomedical analyses: 2018 update. Nucleic Acids Res. 2018;46(W1):W537–44. 10.1093/nar/gky379 PubMed https://doi.org/10.1093/nar/gky379

8. Zhang Z, Schwartz S, Wagner L, Miller W. A greedy algorithm for aligning DNA sequences. J Comput Biol. 2000;7:203–14. PubMed https://doi.org/10.1089/10665270050081478

9. Morgulis A, Coulouris G, Raytselis Y, Madden TL, Agarwala R, Schäffer AA. Database indexing for production MegaBLAST searches. Bioinformatics. 2008;24:1757–64. PubMed https://doi.org/10.1093/bioinformatics/btn322

10. Martin I, Pfyffer GE, Parrish N. *Mycobacterium*: general characteristics, laboratory detection, and staining procedures [chapter]. In: Manual of clinical microbiology. 12th edition. Carroll KC, Pfaller MA, Landry ML, McAdam A, Patel R, Richter SS, et al. (editors). Washington: ASM Press; 2019. p. 562–568.

11. Caulfield AJ, Richter E, Brown-Elliott BA, Wallace Jr RJ, Wengenack NL. *Mycobacterium*: laboratory characteristics of slowly growing mycobacteria other than *Mycobacterium tuberculosis*. In: Manual of clinical microbiology. 12th edition. Carroll KC, Pfaller MA, Landry ML, McAdam A, Patel R, Richter SS, et al. (editors). Washington: ASM Press. 2019; 2019. p. 603–604
